# Microencapsulated phages show prolonged stability in gastrointestinal environments and high therapeutic efficiency to treat *Escherichia coli* O157:H7 infection

**DOI:** 10.1186/s13567-021-00991-1

**Published:** 2021-09-14

**Authors:** Hanjie Yin, Jing Li, Haosheng Huang, Yuxin Wang, Xinjie Qian, Jianluan Ren, Feng Xue, Jianjun Dai, Fang Tang

**Affiliations:** 1 MOE Joint International Research Laboratory of Animal Health and Food Safety, Key Lab of Animal Bacteriology, Ministry of Agriculture, College of Veterinary Medicine, Nanjing Agricultural University, Nanjing, 210095 China; 2 China Pharmaceutical University, Nanjing, China

**Keywords:** *E. coli* O157:H7, microencapsulated phage, antibiotic resistance, therapeutic efficiency

## Abstract

*Escherichia coli* (*E. coli*) O157:H7 bacterial infection causes severe disease in mammals and results in substantial economic losses worldwide. Due to the development of antibiotic resistance, bacteriophage (phage) therapy has become an alternative to control O157:H7 infection. However, the therapeutic effects of phages are frequently disappointing because of their low resistance to the gastrointestinal environment. In this study, to improve the stability of phages in the gastrointestinal tract, *E. coli* O157:H7 phages were microencapsulated and their in vitro stability and in vivo therapeutic efficiency were investigated. The results showed that compared to free phages, the resistance of microencapsulated phages to simulated gastric fluid and bile salts significantly increased. The microencapsulated phages were efficiently released into simulated intestinal fluid, leading to a better therapeutic effect in rats infected with *E. coli* O157:H7 compared to the effects of the free phages. In addition, the microencapsulated phages were more stable during storage than the free phages, showing how phage microencapsulation can play an essential role in phage therapy.

## Introduction

Enterohemorrhagic *E. coli* (EHEC) O157:H7, which produces Shiga toxin, can cause haemorrhagic colitis, bloody diarrhoea, thrombocytopenic purpura and haemolytic uraemic syndrome in humans [1–3]. As a major foodborne pathogen, *E. coli* O157:H7 has become a global public health problem. Although the safety of various products is ensured by implementing control strategies, outbreaks still occur. Thus, it has been estimated that 63 153 cases of foodborne disease and 61 deaths are attributed to *E. coli* O157:H7 annually in the United States, with approximately $255 million in losses each year [4, 5].

*E. coli* is sensitive to the most commonly used antibiotics, but the overuse and misuse of antibiotics have created a global antibiotic resistance crisis [6]. According to one estimation, there are 2.8 million cases of antibiotic-resistant infections and 35 000 deaths in America each year [7]. China is one of the largest countries that produces and uses antibiotics worldwide, and its per capita use of antibiotics is five times greater than that in Europe and America [8]. Nearly half of all antibiotics are discharged into the soil and water, causing pollution and resulting in more than 80 000 deaths each year due to antibiotic resistance [9]. In addition, broad-spectrum antibiotics might put patients at a risk of other intestinal infections due to resident intestinal microbiota dysbiosis [10, 11]. Phages are viruses that specifically infect bacterial cells. Among them, lytic phages can replicate and proliferate rapidly after infecting the host bacteria, finally rupturing the bacterial cells to achieve their antibacterial effect. Compared with traditional antibiotic therapy, phages have the advantages of strong specificity, self-replication, proliferation, safety, no residual effects and plentiful sources, so phage therapy is considered an ideal alternative to antibiotics [12, 13].

Phages are very effective and have been successfully used to prevent and treat bacterial infections in animals [14–16]. Although phages can be administered in a variety of ways, including via parenteral, topical, oral and inhaled routes, by lavage and through eye and nose drops [13, 17], the current problem is that phages lack suitable dosage forms. In particular, during the treatment of animal intestinal diseases, the activity of oral phages is easily destroyed by gastric acid, digestive enzymes and bile salts (BSs) [18, 19], resulting in the loss of antibacterial activity. In addition, their relatively short storage time limits their applications in the fields of food and medicine.

Microencapsulation technology is a new technology that has developed and matured showing wide application potential. This technology that uses natural or synthetic polymers as the encapsulation material to encapsulate the core material (solid, liquid or gas) to form a semipermeable or sealed capsule membrane. The substance that encapsulates the core is the wall material, and the encapsulated product is the core material. In microencapsulation technology, polymers are mainly used to encapsulate functional, active substances and form a dense physical barrier around the core material to reduce the reaction of the core material with the external environment. Microencapsulation of active substances can improve their physical properties and stability, prevent or reduce their deactivation, and prolong their storage time [20]. Ma et al. [21] used the microencapsulated phage Felix O1 for oral administration using a chitosan-alginate-CaCl_2_ system and then detected the activity of free phage and microencapsulated phage in simulated gastric fluid, bile and intestinal fluid. Colom et al. [22] compared the therapeutic effects of orally administered sodium alginate/CaCO_3_-coated phages and free phages in commercial chicken broilers infected with *Salmonella*. Both of these studies demonstrated that microencapsulation significantly improved the survival rate of the phages in the gastrointestinal tract compared with free phages.

Although there have been several studies on the microencapsulation of phages, most of them evaluate the phage microcapsules in vitro, and little is known about the survival of phages in vivo. In this study, phages targeting *E. coli* O157:H7 were microencapsulated by extrusion using the sodium alginate/CaCl_2_ system, and the effects of the microencapsulated phages both in vitro and in vivo were evaluated.

## Materials and methods

### Bacteria and phage

Phage PNJ1901 was isolated from chicken faeces and stored in a lab at Nanjing Agricultural University. *E. coli* O157:H7 was obtained from the Jiangsu Center for Disease Control and Prevention.

### Bacterial culture and phage propagation

*E. coli* O157:H7 were cultured in LB medium at 37 °C in an incubator shaker at 180 rpm. The double-layer agar method was used for phage propagation as follows. First, 100 µL of *E. coli* O157:H7 in the logarithmic phase was mixed with 100 µL of phage PNJ1901. Then, the mixture was added to 5 mL of warm LB medium with 0.5% agar and spread onto an LB plate. The prepared plate was cooled at room temperature for 10 min, followed by incubation at 37 °C for approximately 8 h. After that, 5 mL of SM buffer was added to the double agar plate and incubated at 4 °C overnight. The phage solution was obtained by filtering the SM buffer through a 0.22 μm sterile filter.

### Preparation of microcapsules

Microcapsules were prepared according to Ma’s descriptions with some modifications [21]. First, the designated amount of sodium alginate (Macklin, China) per the experimental protocol was mixed and stirred with ultrapure water to cause the sodium alginate to fully absorb water and swell for use. Second, the designated amount of phage was mixed with sodium alginate. Then, small droplets of the sodium alginate and phage mixture were pushed through a syringe at a uniform speed into a beaker containing cool CaCl_2_ solution on a magnetic stirrer (Thermo Scientific, USA). Finally, the droplets were allowed to solidify in the CaCl_2_ solution to obtain microcapsules, which were washed and filtered repeatedly with distilled water until there was no residual CaCl_2_ on the surface. The microcapsules obtained were stored at 4 °C in sealed tubes. To determine the titre of the microencapsulated phage, a total of 1 g of microcapsules was added to 9 mL of microcapsule-broken solution (MBS) containing 0.2 M sodium bicarbonate, 50 mM sodium citrate and 50 mM Tris–HCl (pH 7.5). The microcapsules were then dissolved with shaking at room temperature for 30 min [21, 23, 24]. Afterwards, 100 µL samples were collected and diluted ten-fold, and the phage titre in the solution was determined by double-layer agar. The phage encapsulation efficiency was calculated as follows: encapsulation efficiency (%)  =  (quantity of phage released from the dissolved microcapsules/quantity of phage initially placed in the prepared microcapsules)  ×  100% [21]. The results are presented as the mean encapsulation efficiency  ±  standard deviation (SD) of the microcapsule preparations. The experiment was repeated three times.

### Orthogonal design for phage microencapsulation analysis

To find a preparation method with high encapsulation efficiency and investigate the optimal parameters of the microencapsulation procedure, five main factors were examined: the concentration of sodium alginate (Factor A, set at 1%, 1.5%, 2%, 2.5% and 3%); the ratio of phage to sodium alginate (Factor B, set at 4:1, 2:1, 1:1, 1:2 and 1:4); syringe needle diameter (Factor C, set at 0.5 mm, 0.6 mm, 0.7 mm, 0.9 mm and 1.2 mm); the concentration of CaCl_2_ (Factor D, set at 1%, 2%, 3%, 4% and 5%), and the speed of stirring (Factor E, set at 0 rpm, 100 rpm, 200 rpm, 300 rpm and 500 rpm) (Table 1). All of the factor levels were set according to consulted references and empirical experience. In addition, there was no interaction between each factor and level. Therefore, a five-factor five-level orthogonal table was designed using SPSS 19.0 (IBM, USA). The assay was repeated three times.

**Table 1:** **Parameters of the orthogonal tests for phage microencapsulation**

No.	A (%)	B	C (mm)	D (%)	E (rpm)	EE (%)
1	3	1:2	1.2	3	0	26.710 ± 1.191
2	2	1:1	0.5	3	500	32.280 ± 0.481
3	1	1:2	0.9	2	500	29.700 ± 1.018
4	1.5	2:1	0.5	2	200	41.057 ± 1.592
5	2.5	4:1	0.7	3	200	30.753 ± 1.005
6	1	4:1	0.5	1	0	55.423 ± 4.356
7	2	4:1	0.9	4	300	30.660 ± 0.407
8	1.5	1:1	0.6	4	0	34.180 ± 1.374
9	1.5	1:2	0.7	1	300	48.633 ± 1.653
10^a^	3	4:1	0.6	2	100	77.767 ± 2.636
11	2.5	2:1	0.9	5	0	27.995 ± 1.270
12	3	1:1	0.9	1	200	34.028 ± 1.867
13	1.5	1:4	0.9	3	100	24.120 ± 1.559
14	2.5	1:1	1.2	2	300	25.707 ± 1.494
15	1.5	4:1	1.2	5	500	38.097 ± 1.962
16	2	2:1	1.2	1	100	55.140 ± 1.702
17	2	1:2	0.6	5	200	30.917 ± 0.820
18	3	1:4	0.5	5	300	26.576 ± 1.134
19	2.5	1:2	0.5	4	100	29.537 ± 1.788
20	2.5	1:4	0.6	1	500	29.930 ± 0.853
21	3	2:1	0.7	4	500	59.243 ± 2.671
22	1	1:1	0.7	5	100	25.440 ± 0.421
23	1	2:1	0.6	3	300	44.530 ± 2.071
24	1	1:4	1.2	4	200	27.153 ± 1.940
25	2	1:4	0.7	2	0	29.290 ± 1.095

A: concentration of sodium alginate; B: ratio of phage to sodium alginate; C: syringe needle diameter; D: concentration of CaCl_2_; E: speed of stirring; EE: encapsulation efficiency (mean ± SD, *n* = 3).

^a^Preparation parameters with the highest encapsulation efficiency.

### Morphology of the microencapsulated phages

The size and surface morphology of the microcapsules were examined by optical microscopy (Leica DM500, Germany).

### Stability of the free and microencapsulated phages in simulated gastric fluid (SGF)

SGF was composed of 0.2% (wt/vol) NaCl and 3.2 mg/mL pepsin (Sigma–Aldrich, USA), with pH values of 2.0 and 2.4 [21]. For the free phage, 100 µL of free phage was mixed with 9.9 mL of prewarmed SGF (pH 2.0 or 2.4) and incubated with shaking at 100 rpm and 37 °C. At each time point (0, 5, 15, 30 and 60 min), a 100 µL sample was collected, ten-fold dilution was made, and the phage titre was determined with double-layer agar. For the microencapsulated phages, 1 g of microencapsulated phage was added to 10 mL of prewarmed SGF (pH 2.0 or 2.4) and incubated at 37 °C with shaking at 100 rpm. At 0, 15, 30, 60, 90, 120, 150, 180 and 240 min, the SGF was aspirated, and 10 mL of SM buffer (pH 7.5) was immediately added to terminate the reaction. The samples were collected, the microencapsulated phage was dissolved at room temperature to release the free phage into MBS as previously described, and the phage titre in the solution was quantified. SM buffer was used as a control. The experiment was repeated three times.

### Stability of free and microencapsulated phages in BSs

BS solution consisted of 1% or 2% (wt/vol) porcine bile extract from Sigma–Aldrich [21]. For the free phages, 100 µL of free phage was placed into 9.9 mL of prewarmed BS and incubated with constant agitation at 100 rpm and 37 °C. Afterwards, a 100-µL sample was added to 900 µL of SM buffer at each predetermined time point (0, 15, 30, 45, 60, 90, 120, 150 and 180 min), and the phage titre was determined by double-layer agar.

For the microencapsulated phages, 1 g of microcapsules was added to test tubes containing 10 mL of prewarmed BS and incubated with constant agitation at 100 rpm at 37 °C. At 0, 15, 30, 45, 60, 90, 120, 150 and 180 min, samples were collected and washed with SM buffer three times, and the microcapsules were cracked to release the free phages into MBS to determine the phage titre. Distilled water (pH 7.0) was used as a control. This experiment was repeated three times.

### Stability of the free phages and release rate of the microencapsulated phages in simulated intestinal fluid (SIF)

SIF was composed of 10 mg/mL pancreatin (Sigma–Aldrich) in 50 mM KH_2_PO_4_ at pH 6.8 [21]. To determine the stability of the free phages in SIF, 100 µL of free phage was mixed with 9.9 mL of prewarmed SIF and incubated with shaking at 100 rpm and 37 °C. At each time point (0, 1, 2, 3, 4, 5, 6, 12 and 24 h), a 100 µL sample was collected, tenfold dilution was made, and phage titre was determined by double-layer agar. To quantify the release rate of the microencapsulated phages, 1 g of microencapsulated phage was placed into conical tubes containing 50 mL of prewarmed SIF and incubated with constant agitation at 100 rpm and 37 °C. At each time point (0, 1, 2, 3, 4, 5, 6, 12 and 24 h), a 100 µL aliquot was removed, and the phage titre was determined after appropriate gradient dilution. The volume of the withdrawn sample was replaced by the same volume of fresh medium. The assay was carried out in triplicate.

### Evaluation of the therapeutic effects of microencapsulated phages in vivo

Female Sprague–Dawley rats (200  ±  20 g) were purchased from SPF Biotechnology Co., Ltd., (China). The rats were allowed free access to water and feed for the first week for acclimation. The rats were then randomly divided into four groups (10 rats/group) as follows: negative control group, positive control group, free phage group and microencapsulated phage group. Streptomycin (5 g/L) was fed to all rats for 24 h to remove the intestinal flora, followed by food and water fasting for 24 h. Then, the rats were pretreated with 1 mL of 5% NaHCO_3_ to neutralize the gastric acid. After 20 min, each rat in the positive control group, free phage group and microencapsulated phage group was administered 2 mL of *E. coli* O157:H7 (10^9^ CFU/mL, 1 mL/100 g) by gavage, while rats in the negative control group received 2 mL of PBS. The concentration of *E. coli* O157:H7 was determined in preliminary experiments. Eight hours after intragastric administration, each rat in the free phage group, microencapsulated phage group and positive control group was given 1 mL of free phage (10^8^ PFU/rat), 1 g of microencapsulated phage (10^8^ PFU/rat), or 1 mL PBS, respectively. During the week after administration, locomotor activity, clinical manifestations, food intake, weight change and death of the rats were observed and recorded; blood was collected to detect the levels of white blood cells (WBCs) and inflammatory markers (TNF-α, IL-6, IL-1β) (Abmart, China); and faeces were collected to detect the concentrations of *E. coli* O157:H7 and phage. *E. coli* O157:H7 in faeces was detected using sorbitol MacConkey agar plates (Hepobiol, China) and a PCR method (*rfbE* and *fliC* were used as specific target genes; Table 2) [25, 26].

**Table 2:** **Primers for PCR detection of *E. coli* O157:H7 in rat faeces**

Primer	Primer sequence (5′–3′)	Target gene	Annealing temperature (°C)	Product size (bp)
O157-F	AAGATTGCGCTGAAGCCTTTG	*rfbE*	55	497
O157-R	CATTGGCATCGTGTGGACAG	*rfbE*	55	497
H7-F	GCGCTGTCGAGTTCTATCGAGC	*fliC*	55	625
H7-R	CAACTGTGACTTTATCGCCATTCC	*fliC*	55	625

### Stability of the microencapsulated phages during storage

Microencapsulated phages were packed in sterile centrifuge tubes and stored at 4 °C. Microencapsulated samples were withdrawn, and the titre was determined each week. Free phages were used as a control.

### Statistical analysis

A five-factor five-level orthogonal table was designed using SPSS version 19.0 for Windows. Statistical analyses were performed using the GraphPad Prism software package (GraphPad Software, USA). Significant differences were evaluated using Student’s *t* test for single group comparisons and one-way ANOVA for multiple comparisons. Figures show mean values  ±  standard deviations. Differences were considered significant at *P * <  0.05.

## Results

### Optimum parameters for phage microencapsulation

The results of the orthogonal experiments showed that the highest encapsulation efficiency of 77.767  ±  2.636% was achieved when the concentration of sodium alginate was 3%, the ratio of phage to sodium alginate was 4:1, the needle diameter of the syringe used for extrusion dropping was 0.6 mm, the concentration of CaCl_2_ was 2%, and the speed of stirring was set at 100 rpm (Table 1).

### The appearance and microstructure of the microencapsulated phages

**Figure 1:**
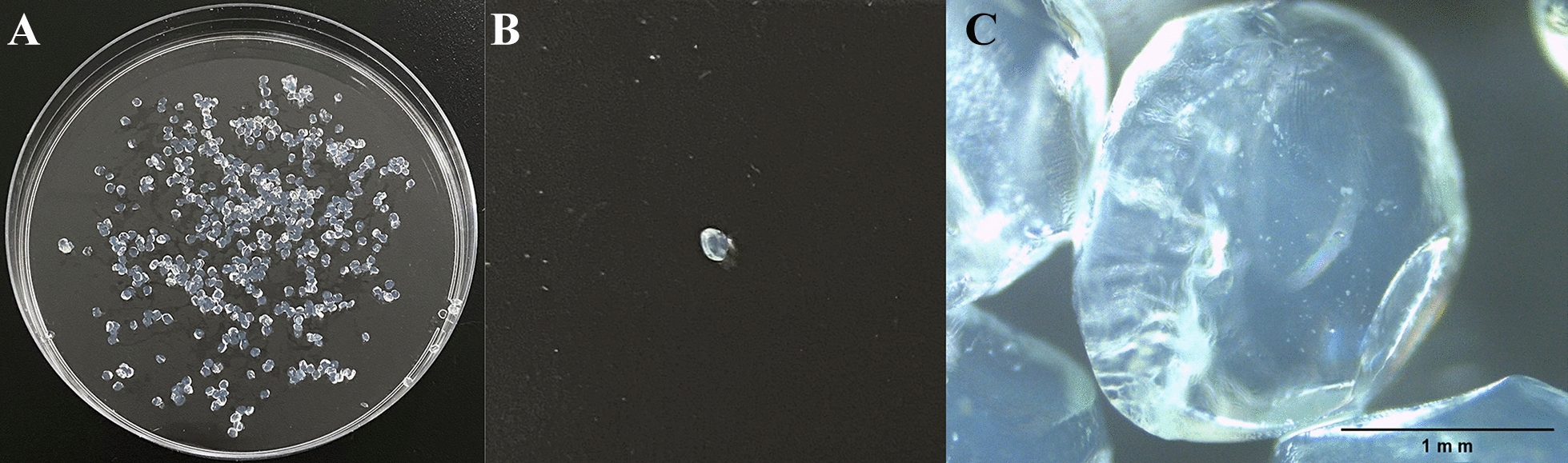
**Images of the microcapsules. A** Physical image of microencapsulated phages. **B** Physical image of a single microencapsulated phage. **C** Optical micrograph of microencapsulated phages.

The appearance of the microencapsulated phages was uniform, with a shape close to that of a sphere (Figure 1A, B). When observed under a microscope, the surface of the microcapsule was nearly smooth with a few slight wrinkles. The average size of the microencapsulated phage particles was 1.759 mm (range 1.746–1.772 mm; Figure 1C).

### Microencapsulated phages are more resistant to SGF than free phages

**Figure 2:**
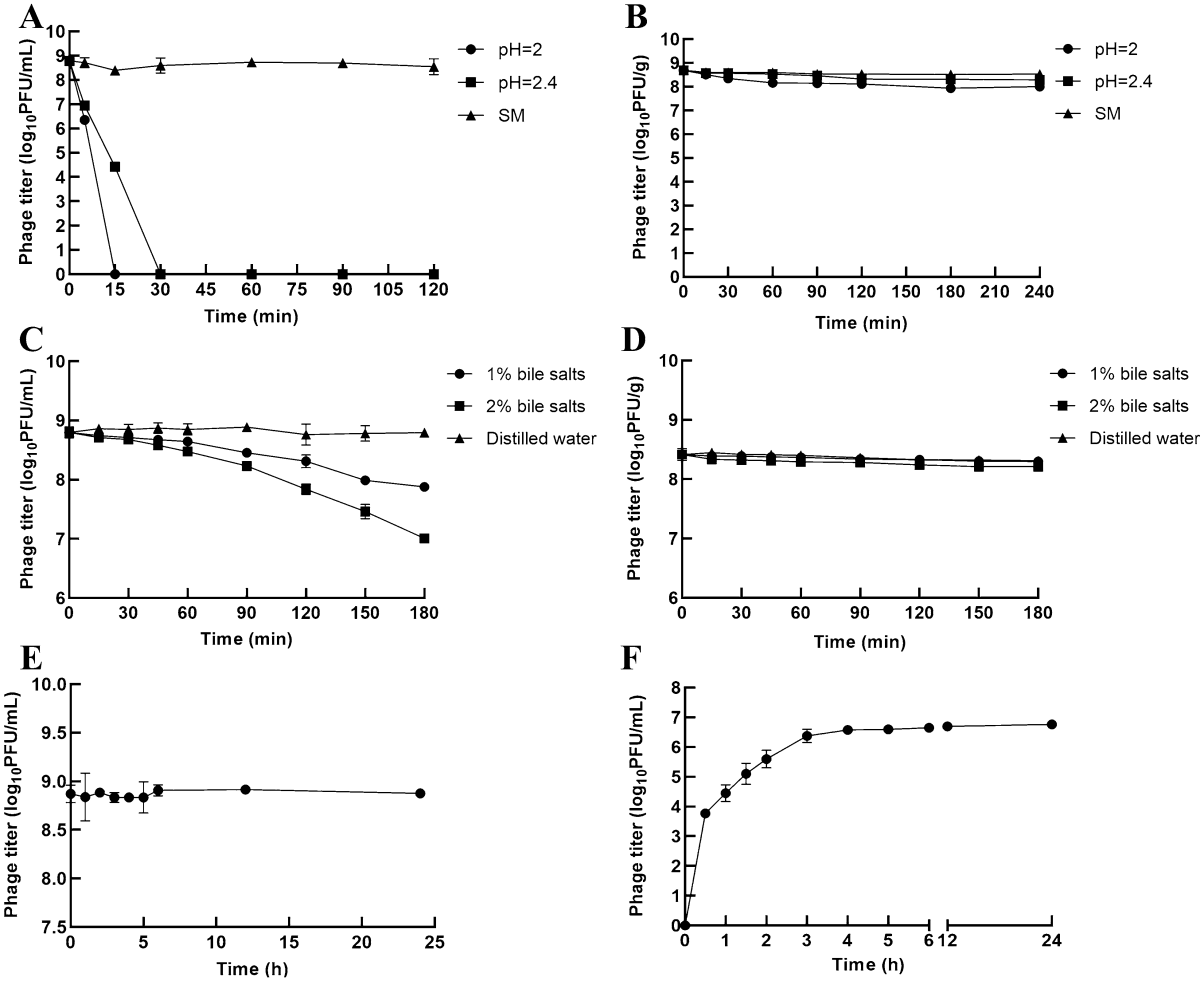
**Stability of microencapsulated phages under simulated gastrointestinal conditions.** Effects of SGF on **A** free phages and **B** microencapsulated phages. Stability of **C** free phages and **D** microencapsulated phage in BSs. Stability of **E** free phages and **F** the release of microencapsulated phages in SIF. Each value in the figure represents the mean  ±  SD (*n * =  3). The stability of the free and microencapsulated phages in SGF, BSs or SIF and the release rate of the microencapsulated phages in SIF were tested by calculating the titre of the phages at different time points after incubation in SGF, BSs or SIF. Each assay was carried out in triplicate.

As the activity of free phages is easily destroyed by gastric acid, we hypothesized that microencapsulated phages would have increased stability in SGF. To test this hypothesis, both free and microencapsulated phages were exposed to SGF. The results showed that the free phage PNJ1901 was sensitive to this acidic environment (Figure 2A) and was mostly inactivated after 15 min or 30 min of incubation in SGF at pH 2 or pH 2.4 (Figure 2A). In contrast to the free phages, microencapsulated phages were quite stable in SGF. After incubation in SGF at pH 2.4 or pH 2 for 240 min, the titres of the microencapsulated phages decreased by only 0.242  ±  0.049 log_10_PFU/g and 0.528  ±  0.053 log_10_PFU/g, respectively (Figure 2B).

### Microencapsulated phages are more resistant to BSs than the free phages

After passing through gastric acid, orally administered phages need to pass through the duodenum with a high concentration of bile acids, so we next determined the stability of the microencapsulated phages in BSs. The titres of the free phages decreased by only 0.202  ±  0.124 log_10_ PFU/mL and 0.371  ±  0.109 log_10_ PFU/mL after incubation for 60 min in 1% and 2% BSs, respectively, while after 180 min of incubation, the titres decreased by 0.915  ±  0.022 log_10_ PFU/mL and 1.787  ±  0.029 log_10_ PFU/mL, respectively (Figure 2C). These results indicated that BSs had little short-term effects on free phages but did great harm after long exposure. The titres of the microencapsulated phages decreased by only 0.012  ±  0.012 log_10_ PFU/g and 0.099  ±  0.058 log_10_ PFU/g in 1% and 2% BSs, respectively, after 180 min of incubation (Figure 2D), indicating that the microencapsulated phages were more stable in BSs compared with the free phages.

### Microencapsulated phages are efficiently released into SIF

Microencapsulated phages not only need to maintain their activity in gastric acid and bile acid but also require quick release in the intestinal tract to clear the target pathogenic bacteria efficiently. Therefore, we subsequently tested the release rate of the microencapsulated phages into SIF. To exclude the effects of SIF on the titre of the released phages, we first detected the stability of the free phages in SIF, and the results showed that the titre of the free phages remained stable without significant loss after 24 h of incubation (Figure 2E). Thus, the free phages had good tolerance to SIF. In the next release experiment, we observed that the release of the phages from the microcapsules in SIF increased with time. After incubation in SIF for 6 h, the titre of the released phages was 6.653  ±  0.020 log_10_ PFU/mL, suggesting a cumulative release of 70.313% of the starting titre (Figure 2F). After incubation in SIF for 24 h, the titre of the released phages reached 6.761  ±  0.012 log_10_ PFU/mL, representing a release rate of 90.104%, which was close to complete release (6.806 log_10_ PFU/mL) (Figure 2F).

### Microencapsulated phages show better therapeutic effects than free phages in vivo

**Figure 3:**
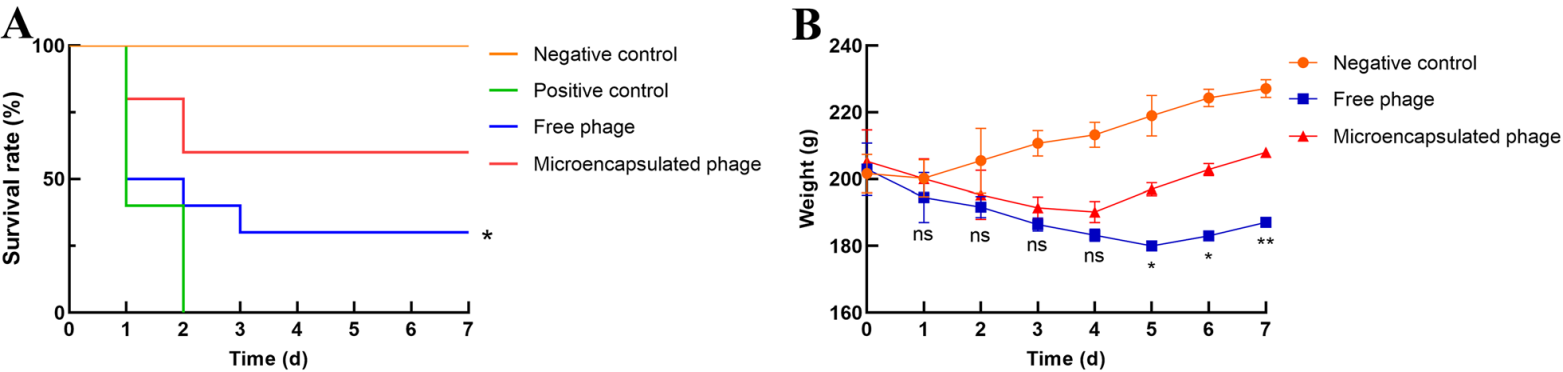
**Therapeutic effects of microencapsulated phages in vivo**. **A** Survival curves of rats within one week after administration of *E. coli.* Rats challenged with *E. coli* O157:H7 (10^9^ CFU/mL, 1 mL/100 g) were gavaged with free or microencapsulated phages (10^8^ PFU/rat) 8 h after infection. Rats in the positive control group were gavaged with *E. coli* O157:H7 and PBS. Rats in the negative control group received only 2 mL of PBS. Survival rates of the rats in all groups were monitored. **B** Changes in the weights of the rats within one week after administration of *E. coli*. The weights of the rats in the free phage, microencapsulated phage and negative control groups were monitored for 1 week. Statistical analysis was used to compare the microencapsulated phage group and the free phage group. ns, no significant difference; *P  <  0.05; **P  <  0.01.

Since the microencapsulated phages performed better than free phages in vitro, we next tested their effects in vivo. Rats infected with *E. coli* O157:H7 received microencapsulated or free phages 8 h post-infection. Rats receiving *E. coli* or PBS alone were used as positive and negative controls, respectively. The rats were monitored for 7 days. In the free phage group, the rats were less active, huddled together and ate less in the first 5 days after infection, but their condition improved during the last 2 days. Rats in the microencapsulated phage group had similar symptoms as those in the free phage group, but they began to recover on Day 4, which was 2 days earlier than the free phage group. To assess survival, 30% of rats in the free phage group survived, while the survival rate of the rats in the microencapsulated phage group increased to 60% (*P*  <  0.05; Figure 3A). In contrast, all of the rats receiving *E. coli* alone died, while the rats in the negative control group receiving PBS all survived (*P*  <  0.05; Figure 3A).

**Figure 4:**
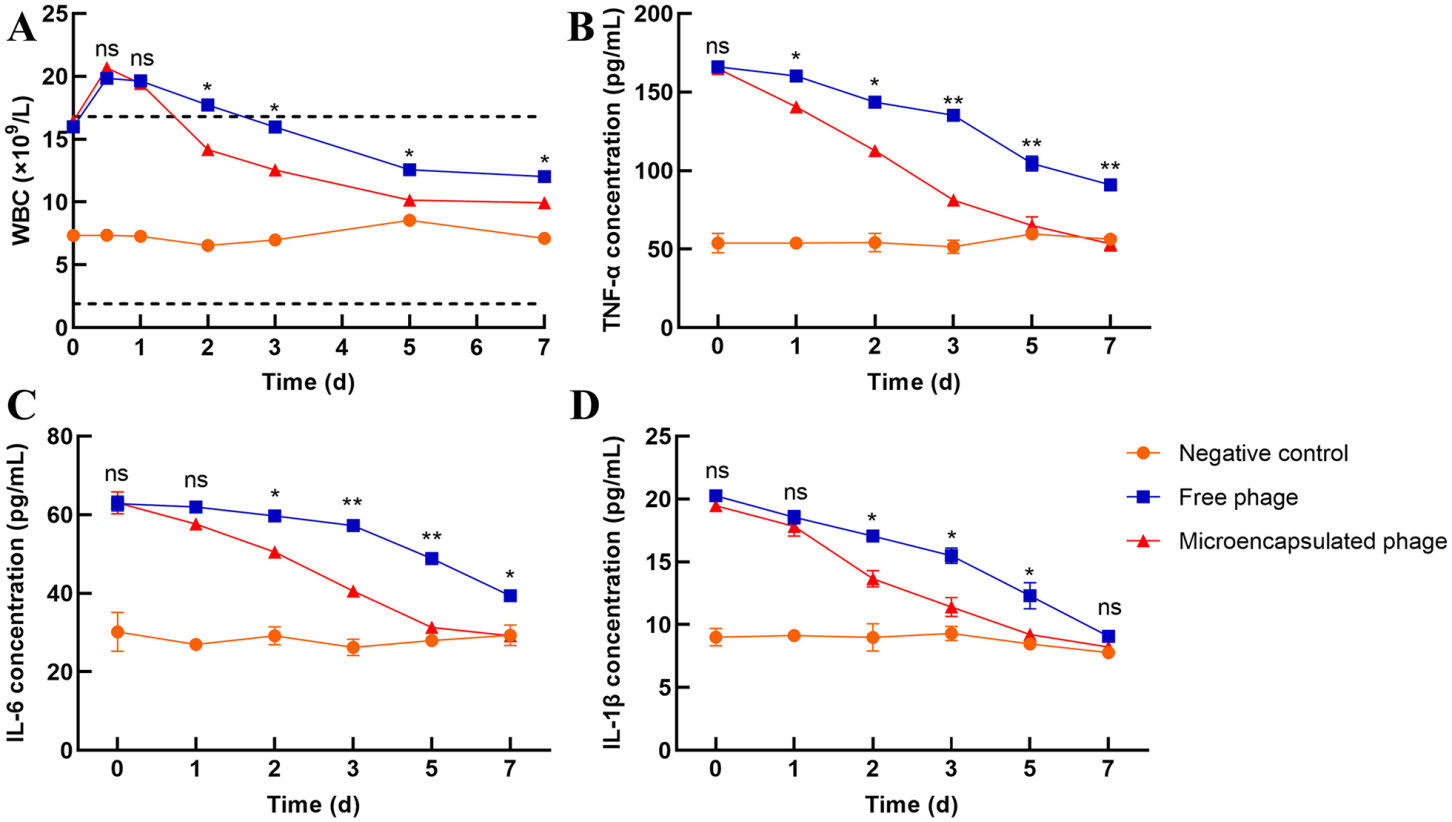
**The concentration of WBCs and inflammatory factors in vivo. A** Changes in WBCs in rats within 1 week after administration of *E. coli*. The two dashed lines represent the normal range of WBCs in rats. Changes in **B** TNF-α, **C** IL-6 and **D** IL-1β in rats within one week after administration of *E. coli*. Each value in the figure represents the mean  ±  SD (*n*  =  3) with the exception of Figure 4A. Statistical analysis was used to compare the microencapsulated phage group and the free phage group. ns, no significant difference; *P  <  0.05; **P  <  0.01.

As shown in Figure 3B, the rats in the negative control group gained weight continuously throughout the week of observation. The weights of the rats in both the free phage group and microencapsulated phage group first decreased and then increased. However, the weights of the rats in the microencapsulated phage group increased earlier and to a greater extent (*P*  <  0.05) than the weights of the rats in the free phage group, which was consistent with the behavioural symptoms, indicating that the rats in the microencapsulated phage group recovered faster and more completely than the rats in the free phage group. The fluctuation in WBC count and TNF-α, IL-6 and IL-1β content in rat blood during the first week after infection were also monitored. As shown in Figure 4A, the WBC counts of the rats in the negative control group were in the normal range. The WBC counts in the free phage group were higher than the normal range during the first 2 days, then decreased to the normal range and continued to decline but were still higher than the counts in the negative control group. The WBC counts in the microencapsulated phage group decreased to the normal range after the first day, displaying a faster decrease than that of the free phage group. By Day 7, the WBC counts were closer to those of the negative control group than to those of the free phage group. As shown in Figure 4B–D, the changes in TNF-α, IL-6 and IL-1β contents were similar in both phage groups. Although the cytokines in both the free phage group and microencapsulated phage group were initially much higher than those in the negative control group, they continued to decline during the first week after infection. Cytokines in the microencapsulated phage group decreased faster (*P * <  0.05) than in the free phage group, dropping to nearly the same level as the negative control group on Day 5, while the concentrations of cytokines in the free phage group remained higher than the negative control group on Day 7, indicating that the microencapsulated phages had a more potent effect on inflammation.

**Figure 5:**
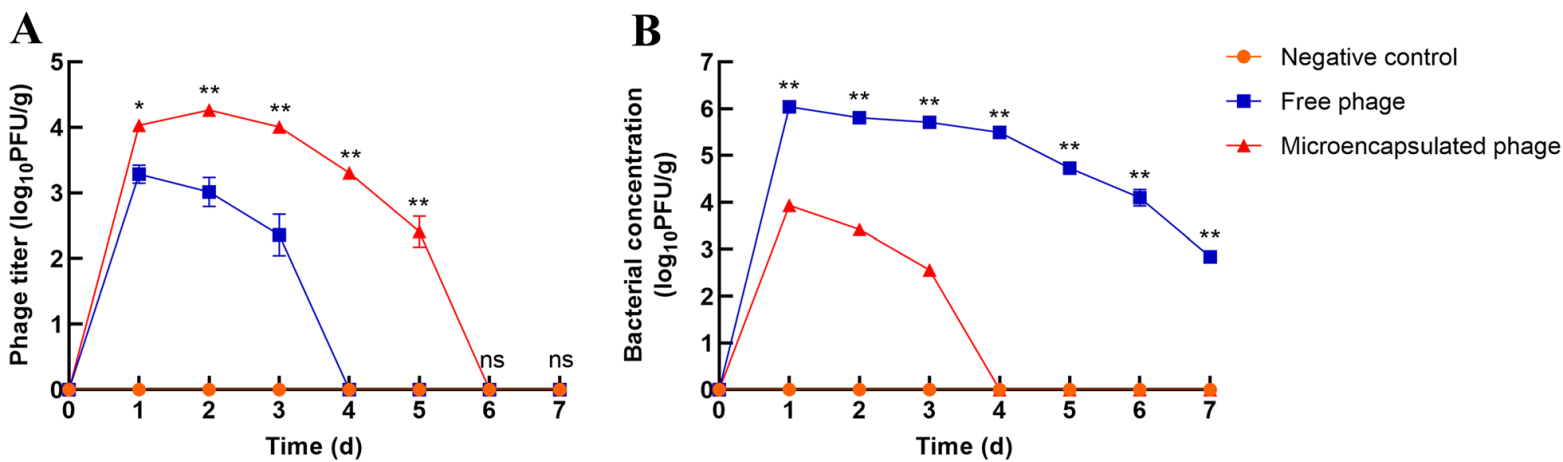
**Phage and bacterial load in faeces. A** Phage titre in the faeces of rats within 1 week after administration of *E. coli*. **B** Bacterial concentration in the faeces of rats within 1 week after administration of *E. coli*. Faeces of rats from the free phage, microencapsulated phage and negative control groups were collected to detect the concentrations of *E. coli* O157:H7 and phage. *E. coli* O157:H7 in faeces was detected using sorbitol MacConkey agar plates and the PCR method (*rfbE* and *fliC* were used as specific target genes). Phage titre was determined by double-layer agar. Each value in the figure represents the mean  ±  SD (*n * =  3). Statistical analysis was used to compare the microencapsulated phage group and the free phage group. ns, no significant difference; *P  <  0.05; **P  <  0.01.

To determine whether the better therapeutic effects of the microencapsulated phages were due to their stability in vivo, the concentrations of phages and bacteria in the faeces of rats was tested for one week after infection. As shown in Figure 5A, the phage titre in the rat faeces from the microencapsulated phage group was significantly higher (*P * <  0.05) than the titre in the faeces from the free phage group. Additionally, phages were detected for 5 consecutive days in the faeces from the microencapsulated phage group, while phages were not detected at all in faeces from the free phage group on Day 4. *E. coli* O157:H7 in faeces was identified using sorbitol MacConkey agar plates and PCR. As shown in Figure 5B, the bacterial concentration in the faeces from the microencapsulated phage group was significantly lower (*P*  <  0.01) than its concentration in the faeces from the free phage group. *E. coli* O157:H7 was not detected on Day 4 in faeces from the microencapsulated phage group, while it was detected in faeces from the free phage group for 7 consecutive days. These results suggested that the microencapsulated phages were more stable in vivo, which led to the greater bactericidal effects than those of the free phages.

### Microencapsulated phages are more stable than free phages during storage

**Figure 6:**
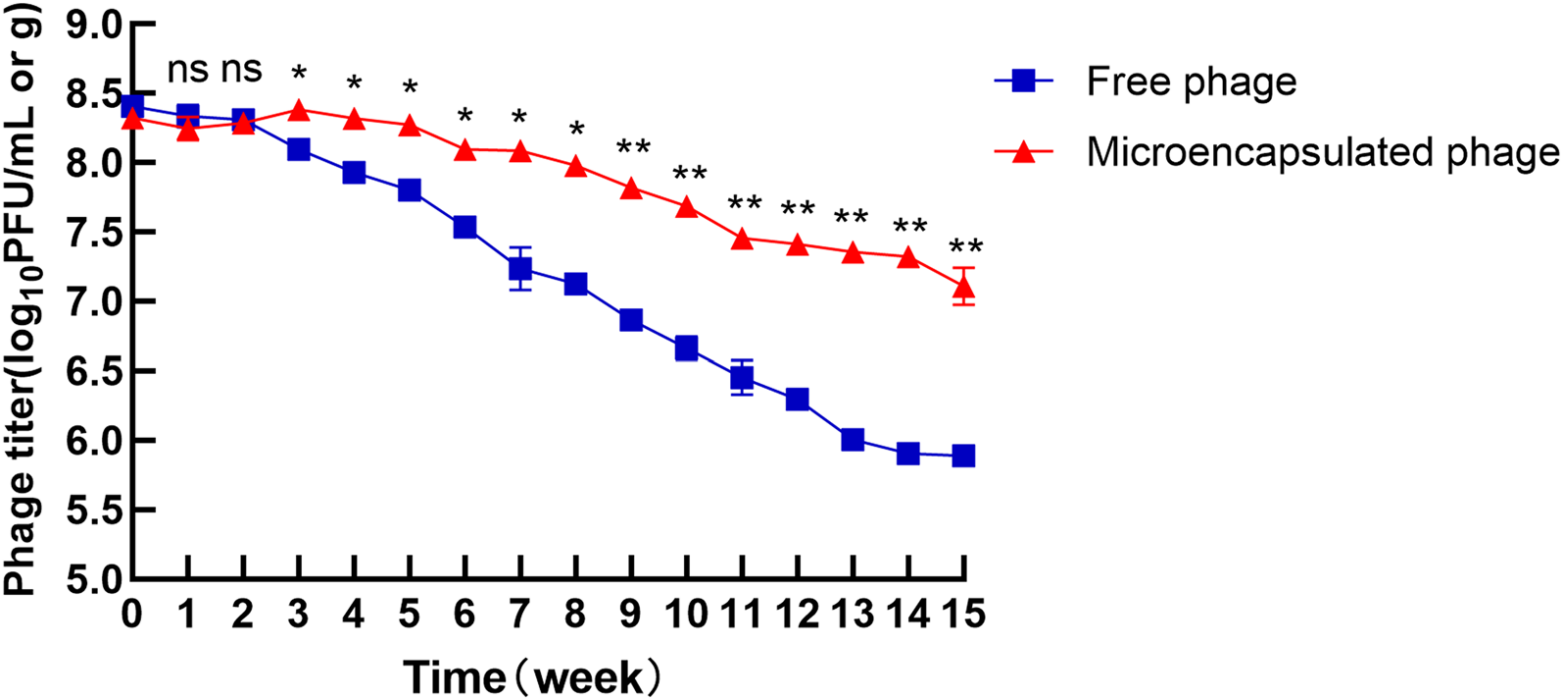
**Stability of free and microencapsulated phages during storage.** Free and microencapsulated phages were stored at 4 °C, and the titre was measured every week. Each value in the figure represents the mean  ±  SD (*n* = 3). Statistical analysis was used to compare the microencapsulated phage group and the free phage group. ns, no significant difference; *P  <  0.05; **P  <  0.01.

Preserving the stability of therapeutic agents during storage is very important, so we compared the stabilities of microencapsulated and free phages during storage. The titre of the free phages decreased nearly 3 log units when it was stored at 4 °C for 15 weeks, while the titre of the microencapsulated phages decreased only 1 log unit when stored for the same period (*P*  <  0.01; Figure 6). These results demonstrated that microencapsulated phages were more stable than free phages when stored at 4 °C.

## Discussion

Viruses (including phages) easily lose their activity in the presence of acid, heat and organic solvents and in the absence of water [21]. Encapsulation technologies are the most common methods to protect phages from adverse conditions. Extrusion dropping methods, spray drying, emulsion and polymerization techniques are the most popular techniques and methods for encapsulating microorganisms [27]. The extrusion dropping method was adopted in this study because it is relatively simple for the production of microcapsules. In addition, the preparation conditions are mild, and the size of the prepared microcapsules is uniform. Cellulose, liposomes, alginate, whey protein and gelatine are the most common biomaterials for phage encapsulation and have been applied by different technologies [27, 28]. Sodium alginate is a good microencapsulation material with good biocompatibility that utilizes a mild gel process and does not show toxicity [21]. In addition, the molecular chain of sodium alginate shrinks at low pH and stretches at high pH, showing clear pH sensitivity that makes it suitable for encapsulating oral phages. Sodium alginate can react with Ca^2+^ to form a gel [23, 29]. The reaction mechanism involves Ca^2+^ and two GG fragments on the molecular chain of sodium alginate interacting through four coordination bonds to form the so-called “egg-box” structure, which causes the adjacent molecular chain of sodium alginate to transform from random line clusters into curved band structures cross-linked by Ca^2+^ that intertwine to form a three-dimensional gel network. The sodium alginate/CaCl_2_ system was used for phage microencapsulation in this study.

The concentration of sodium alginate affects not only the encapsulation efficiency of the microencapsulated phages but also the shape, mechanical strength and difficulty of forming microencapsulated phages [30–33]. When the concentration of sodium alginate is low, it cannot fully react with CaCl_2_, resulting in the formation of a thin microcapsule membrane with a soft texture and an irregular shape that damages easily, leading to a low encapsulation efficiency. The encapsulation efficiency increases with increasing sodium alginate concentration. However, when the concentration of sodium alginate is greater than 3%, the viscosity of the solution becomes too high, making passage through the pinhole of the syringe difficult and leading to atypically shaped microcapsules (with tails). The ratio of phage to sodium alginate affects the retention rate of the phage and the encapsulation efficiency of the microcapsules [34]. Different needle diameters used for extrusion dropping will directly lead to different volumes of extruded droplets. During the process of preparing microcapsules, Ca^2+^ acts as a cross-linking agent, which makes the independent linear molecules connect with each other to form a space network structure that is conducive to the formation of microcapsules [35]. When the concentration of CaCl_2_ is too low, there is not enough Ca^2+^ to cross-link with the Na^+^ in sodium alginate, so the microcapsules are slow to form and show a trailing phenomenon, increased adhesion and a soft texture. With increasing CaCl_2_ concentration, the degree of cross-linking between Ca^2+^ and sodium alginate increases, causing the hardness of the microcapsule to increase. However, when the concentration of CaCl_2_ is too high, the outer layer of CaCl_2_ and sodium alginate solidifies rapidly, forming a dense cross-linked structure before the inner layer solidifies. This makes the capsule wall thicker and brittle and hinders both the continuous diffusion of Ca^2+^ and the full encapsulation of phage into the inner layer of the microcapsule, resulting in a hard microencapsulated phage with low encapsulation efficiency. Stirring speed is also very important because it affects the solidification of the microcapsules. A low stirring speed is not conducive to full phage encapsulation, resulting in low encapsulation efficiency, while high stirring speed will lead to phage loss.

The primary requirement for oral phage therapy to treat intestinal diseases is phage survival in gastric acid, but most free phages are extremely sensitive to low pH conditions and are easily inactivated when the pH value is below 3.5 [36]. A study has shown that solutions and small pellets (less than 2 mm in size) empty from the stomach quite rapidly and are not greatly affected by individual digestive states [37]. The average half-emptying time of liquids and pellet systems in vivo is approximately 0.5–1.5 h [37]. Another study showed that the average passage time through the small intestine in a healthy body is 3–4 h, with a maximum time of 6 h [37]. In the present study, the particle size of the microencapsulated phages was less than 2 mm, so it could be emptied from the stomach in a short time; moreover, sodium alginate can shrink in the stomach to protect the phage from damage caused by gastric acid and digestive enzymes, ensuring that the activity of microencapsulated phage will not be greatly affected at pH 2 or pH 2.4. In contrast, the free phages were completely inactivated within 30 min and clearly could not survive well in gastric acid.

Bile acids are synthesized by the liver and stored in the gallbladder when digestion is not occurring and then are discharged in large quantities from the liver or the gallbladder [38, 39] directly into the duodenum during digestion. After passing through gastric acid, the oral phage needs to pass through the duodenum with a high concentration of bile acids. Our results showed that the stability of phages in bile salts was significantly improved after microencapsulation. Compared with gastric acid, bile salts have less of an effect on the activity of free phages. The reason for this may be that the phages do not contain lipid components, so they are tolerant to amphoteric molecules such as bile salts.

Microencapsulated phages need to not only maintain their activity in gastric acid and bile salts but also be released in the intestinal tract in a timely manner. Nearly 70.313% of the total amount of microencapsulated phages were released into SIF within 6 h, and almost all were released within 24 h, leading to bacterial elimination. We also investigated the stability of the free phage PNJ1901 into SIF before we performed the microencapsulated phage release experiment. Ramirez et al. [40] conducted release experiments of microencapsulated phages in SIF in vitro and found that the release rate was significantly slower than that of phages incubated in PBS. However, due to the lack of stability experiments with the free phages in SIF, they could not determine whether this phenomenon was due to phage release failure or the inactivation of the released phages caused by enzymes in the SIF [40]. In the present study, our results showed that the free phages had good tolerance to SIF, ensuring their stability when the microencapsulated phages were released into SIF.

Based on the superiority of the microencapsulated phages over their free counterparts, we were very interested in the therapeutic effects of the microencapsulated phages in vivo. This experiment was conducted in Sprague Dawley rats. We found that high doses of *E. coli* O157:H7 could lead directly to the death of rats, with obvious pathological changes such as a yellowing and odorous intestinal cavity, enlargement of the stomach, thinning and adhesion of the intestinal wall, and damage to the duodenal villi. We selected 10^9^ CFU/mL *E. coli* O157:H7 as the optimal gavage dose, as this dose caused the death of all rats in our preliminary experiment. In the therapeutic experiment, 5 g/L streptomycin was fed for 24 h to the rats 2 days in advance of *E. coli* exposure to clear the intestinal flora and reduce their later impact. Fasting 1 day in advance allowed the emptying of residual food from the intestinal tract and increased the likelihood of *E. coli* O157 contacting the intestinal mucosa; furthermore, 1 mL of 5% NaHCO_3_ was given by gavage 20 min before *E. coli* challenge, which neutralized gastric acid and reduced the possible damage to *E. coli* O157. The survival curves of the rats during the week after *E. coli* administration thus directly reflected the therapeutic effect. In this study, the survival rates of the rats in the free phage group and microencapsulated phage group were 30% and 60%, respectively, which indicated that the protective effect of the microcapsule was substantial. Moreover, the change in weight within one week after administration of the phages may reflect rat recovery. In this study, the weight gain of the rats in the microencapsulated phage group occurred earlier than the weight gain of the rats in the free phage group, suggesting that the recovery of the rats in the microencapsulated phage group was faster than the recovery of the rats in the free phage group.

TNF-α is a cytokine that can activate and aggregate inflammatory cells and promote the release of a variety of inflammatory mediators, such as IL-6 and IL-1β, in a variety of cells [41]. As proinflammatory cytokines, IL-1β and IL-6 are widely involved in many pathological injury processes, such as tissue destruction and oedema formation. When inflammation occurs in the body, a variety of cells can secrete these cytokines after stimulation. The levels of inflammatory cytokines thus reflect the conditions of body injury and inflammation. In the present study, the levels of TNF-α, IL-6 and IL-1β in the serum of rats were detected by ELISA during the week after bacteria and phage administration. The decrease in cytokines in the microencapsulated phage group occurred earlier and faster than the decrease in the free phage group, indicating that the microencapsulated phage had a greater effect on the elimination of inflammation. As the microencapsulated phages were more resistant to SGF and BSs and showed good release in SIF in vitro, we hypothesized that the greater therapeutic effect was due to the increased stability of the microencapsulated phages in vivo compared to the free phages. Therefore, we examined the titre of the phages and the concentration of bacteria in faeces. The results showed that the titre of the microencapsulated phages was significantly higher and lasted longer than that of the free phages, which led to a lower concentration of pathogenic bacteria in faeces. The higher titre of the phage in faeces was likely due to the prolonged survival of more phages in gastric acid and bile. The long duration of phage titre detection in faeces from the microencapsulated phage group was likely the result of slow phage release from the microcapsules. The results of the above experiments show that the therapeutic effects of microencapsulated phages in vivo are better than those of free phages, indicating that the stability of phages is important for therapeutic efficiency in vivo.

It should be noted that rats were gavaged with streptomycin (5 g/L) to remove the intestinal flora and with 5% NaHCO_3_ to neutralize gastric acid before bacterial infection. This pretreatment aimed to facilitate colonization of *E. coli* O157:H7 in the intestine, but the side effect was that the intestinal microbiota was destroyed. Although the microbiota will recover over time, it will not be the same as that in a truly infected animal with a full microbiome. The gut microbiota has several physiological effects on the host immune system, both locally and systemically, which consequently influences the efficacy of drugs and vaccinations [42, 43]. Future studies should be performed to evaluate the therapeutic effects of microencapsulated phages in truly infected animals.

In summary, in this study, we prepared microencapsulated phages and evaluated their stability in vitro and their therapeutic effects in vivo. We showed that compared to free phages, microencapsulation could protect phages from gastric acid and bile acid and that they could be released in the intestinal tract under simulated gastrointestinal conditions in vitro. In addition, microencapsulated phages had longer stability during storage. More importantly, animal experiments demonstrated that the therapeutic effects of microencapsulated phages in vivo were better than those of free phages. All of these results confirm the feasibility and effectiveness of phage microencapsulation, suggesting that microencapsulated phages have the potential to protect against *E. coli* O157 infections in animals. Although there are still many problems and obstacles in phage therapy, especially in gastrointestinal therapy, phage microencapsulation can improve the resistance of phages in the gastrointestinal tract and enhance their activity in vivo, which may improve the effectiveness of phage therapy as a substitute for antibiotics.

## Data Availability

The datasets used and/or analysed during the current study are available from the corresponding author upon reasonable request.

## Abbreviations

*E. coli*: *Escherichia coli*
EHEC: Enterohemorrhagic *Escherichia coli*
BSs: bile salts
SGF: simulated gastric fluid
SIF: simulated intestinal fluid
WBCs: white blood cells
TNF: tumour necrosis factor
IL: interleukin
MBS: microcapsule-broken solution
SD: standard deviation
